# Supplementary material for: Association between Circulating Levels of 25-Hydroxyvitamin D3 and Matrix Metalloproteinase-10 (MMP-10) in Patients with Type 2 Diabetes
Source: Nutrients. 2022 Aug 24;14(17):3484. doi: 10.3390/nu14173484 (PMC9458174; doi:10.3390/nu14173484)
Supplement: Supplementary file 1 [file nutrients-14-03484-s001.zip › nutrients-1883859-supplementary.pdf]

Supplementary Table S1. Correlation between circulating MMP-10 and 25(OH)D_3 levels using alternative cut-offs

		N	Spearman's rho (CI*)	p-value
**Overall**		**256**	**-0.25 (-0.36; -0.13)**	**< 0.001**
Subgroup analysis by vitD3 status	> 45 ng/mL	13	**-0.73 (-1.10; -0.36)**	**< 0.001**
	30-45 ng/mL	53	**-0.33 (-0.55; -0.11)**	**0.003**
	< 30 ng/mL	190	**-0.29 (-0.42; -0.16)**	**< 0.001**

Abbreviations: CI: confidence interval.

* CI for Spearman's rho coefficient were estimated using bootstrapping method

Supplementary Table S2. Correlation between serum 25(OH)D_3 and MMP-10 levels and other variables

	**25(OH)D3**		**MMP-10**	
**Variable**	**Spearman's rho**	**p**	**Spearman's rho**	**p**
Intact PTH (n=145)	-0.45	< 0.001	0.42	< 0.001
Serum creatinine	-0.35	< 0.001	0.54	< 0.001
Serum cystatin	-0.33	< 0.001	0.58	< 0.001
CKD	-0.30	< 0.001	0.39	< 0.001
UACR (n = 255)	-0.29	< 0.001	0.37	< 0.001
Urea (n = 236)	-0.27	< 0.001	0.43	< 0.001
Urate (n=217)	ns		0.26	< 0.001
Triglycerides (n= 253)	-0.20	0.002	0.22	< 0.001
HbA1c (n = 255)	-0.20	0.002	0.15	0.019
Loop diuretics	-0.18	0.004	0.28	< 0.001
Age	ns		0.25	< 0.001
Diabetes duration	-0.17	0.005	0.19	0.003
CRP (n = 171)	-0.17	0.029	0.26	0.001
CCB (n = 255)	-0.14	0.029	0.17	0.006
HTA	-0.13	0.043	0.17	0.006
Body fat % (CUN-BAE)	0.13	0.040	ns	
Hb (n = 253)	0.18	0.004	-0.24	< 0.001
Sex	0.26	< 0.001	-0.17	0.006
HDL (n = 255)	0.27	< 0.001	-0.23	< 0.001
eGFRcr (CKD-EPI 2021)	0.27	< 0.001	-0.55	< 0.001
VitD supplement	0.37	< 0.001	ns	

Note 1: As there were missing values, the number of patients for which the values were available, are given in the parenthesis.

Note 2: Only the significant (p-value < 0.05) correlations are presented. Negative correlations are marked by red shading, positive correlations are marked by green shading, colour intensity corresponds to the strength of the correlation.

Abbreviations: CCB: calcium channel blockers, CKD: chronic kidney disease, CKD-EPI: Chronic Kidney Disease Epidemiology Collaboration, CRP: C reactive protein, CUN-BAE: Clinica Universidad de Navarra Body Adiposity Estimator, eGFRcr: estimated glomerular filtration rate using creatinine, HDL: high-density lipoprotein,

ns: non-significant correlation, PTH: parathyroid hormone, UACR: urinary albumin-creatinine

Supplementary Figure S1. Correlation between MMP-10 and 25(OH)D_3 stratified by 25(OH)D_3 circulating level

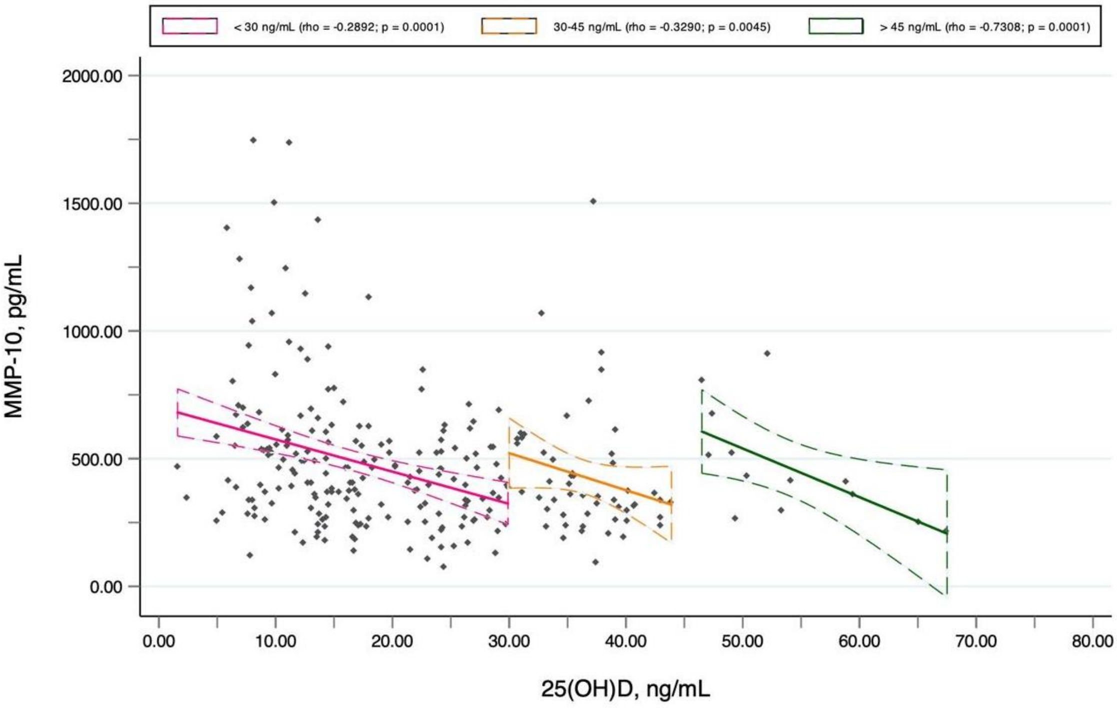

Note: dashed lines show 95% CI
